# Presentation and outcome of Middle East respiratory syndrome in Saudi intensive care unit patients

**DOI:** 10.1186/s13054-016-1303-8

**Published:** 2016-05-07

**Authors:** Ghaleb A. Almekhlafi, Mohammed M. Albarrak, Yasser Mandourah, Sahar Hassan, Abid Alwan, Abdullah Abudayah, Sultan Altayyar, Mohamed Mustafa, Tareef Aldaghestani, Adnan Alghamedi, Ali Talag, Muhammad K. Malik, Ali S. Omrani, Yasser Sakr

**Affiliations:** Department of Intensive Care Services, Prince Sultan Military Medical City, Riyadh, 11159 Saudi Arabia; Intensive Care Unit, Prince Sultan Cardiac Center, Riyadh, 11159 Saudi Arabia; Department of Medicine, King Faisal Specialist Hospital and Research Centre, Riyadh, 11211 Saudi Arabia; Department of Anesthesiology and Intensive Care, Uniklinikum Jena, 07743 Jena, Germany

**Keywords:** Coronavirus, MERS-CoV, Respiratory failure, Epidemic, Saudi Arabia

## Abstract

**Background:**

Middle East respiratory syndrome coronavirus infection is associated with high mortality rates but limited clinical data have been reported. We describe the clinical features and outcomes of patients admitted to an intensive care unit (ICU) with Middle East respiratory syndrome coronavirus (MERS-CoV) infection.

**Methods:**

Retrospective analysis of data from all adult (>18 years old) patients admitted to our 20-bed mixed ICU with Middle East respiratory syndrome coronavirus infection between October 1, 2012 and May 31, 2014. Diagnosis was confirmed in all patients using real-time reverse transcription polymerase chain reaction on respiratory samples.

**Results:**

During the observation period, 31 patients were admitted with MERS-CoV infection (mean age 59 ± 20 years, 22 [71 %] males). Cough and tachypnea were reported in all patients; 22 (77.4 %) patients had bilateral pulmonary infiltrates. Invasive mechanical ventilation was applied in 27 (87.1 %) and vasopressor therapy in 25 (80.6 %) patients during the intensive care unit stay. Twenty-three (74.2 %) patients died in the ICU. Nonsurvivors were older, had greater APACHE II and SOFA scores on admission, and were more likely to have received invasive mechanical ventilation and vasopressor therapy. After adjustment for the severity of illness and the degree of organ dysfunction, the need for vasopressors was an independent risk factor for death in the ICU (odds ratio = 18.33, 95 % confidence interval: 1.11–302.1, *P* = 0.04).

**Conclusions:**

MERS-CoV infection requiring admission to the ICU is associated with high morbidity and mortality. The need for vasopressor therapy is the main risk factor for death in these patients.

**Electronic supplementary material:**

The online version of this article (doi:10.1186/s13054-016-1303-8) contains supplementary material, which is available to authorized users.

## Background

Middle East respiratory syndrome coronavirus (MERS-CoV) is a novel betacoronavirus that was first reported in September 2012 [1]. By January 6, 2016, a total of 1626 laboratory-confirmed cases of infection with MERS-CoV, including at least 586 related deaths, had been reported to the World Health Organization [2]. Although MERS-CoV infections have been reported from 26 countries around the world, the majority of cases have originated in Saudi Arabia, South Korea, the United Arab Emirates, Jordan and Qatar [3]. Inter-human MERS-CoV transmission occurs in community and healthcare settings [4–8]. The exact source and mode of transmission of MERS-CoV to humans remains uncertain. However, MERS-CoV circulates among dromedary camels in Africa and the Middle East with occasions of documented camel-human inter-transmission [9].

There have been several reports outlining the clinical features and outcomes of patients with MERS-CoV infection [7, 10–15]. However, very few have focused on critically ill patients in intensive care units (ICU) [16–18]. There is therefore a need for more data to understand the various clinical and prognostic aspects of this potentially lethal disease, particularly for the most severe cases that require admission to the ICU.

We performed a retrospective study to describe the clinical features and outcomes of patients admitted to our ICU with laboratory-confirmed MERS-CoV infection.

## Methods

### Ethics, consent and permissions

The study was approved by the institutional review board of Prince Sultan Military Medical City (11159 Riyadh, Saudi Arabia), a large tertiary-care referral center in Riyadh, Saudi Arabia. Informed consent was waived due to the retrospective, anonymous nature of data collection. We included all patients aged 18 years or more with confirmed MERS-CoV infection who were admitted to our 20-bed mixed medico-surgical ICU between October 1, 2012 and May 31, 2014.

### Data collection

All ICU patients with a confirmed diagnosis of MERS-CoV were registered in a special logbook. For the purpose of the current study, all patients’ records were reviewed by a senior intensivist (S. Hussain, A. Alwan, A. Abudayah, S. Altayyar, M. Mustafa, T. Aldaghestani, A. Alghamedi, A. Talag or M. Malik). Clinical data and laboratory parameters from confirmed cases of MERS-CoV were transcribed onto specially developed case record forms. These included the initial manifestations of respiratory infection, the clinical picture on admission to the ICU, laboratory indices of organ failure, radiographic findings, interventions during the ICU stay, treatment modalities, and final outcome. The Acute Physiology and Chronic Health Evaluation II (APACHE II) score was calculated from the data obtained within 24 hours of admission to the ICU [19]. The Sequential Organ Failure Assessment (SOFA), score, calculated daily by the physician in charge of the patient, was also noted [20].

### Laboratory investigations

Since the first reported cases of MERS-CoV in Saudi Arabia in September 2012, all suspected cases in our institution are strictly isolated and nasopharyngeal swabs are obtained for initial screening. Deep respiratory samples (tracheal aspirates or bronchoalveolar lavage fluid) are obtained from all patients admitted to the ICU with suspected respiratory infections, in addition to blood samples to perform cultures and polymerase chain reaction for common respiratory viruses and atypical microorganisms. Urinary samples were obtained to detect Legionella antigens in only two patients (Legionella infections are not common in Saudi patients). Cultures of tracheal aspirates are analyzed quantitatively and bacterial counts of at least 10^5^ colony-forming units are considered positive. These investigations are repeated in the ICU whenever secondary infections are suspected. Clinical specimens aimed at detecting possible MERS-CoV infection are processed and analyzed at the National Reference Laboratory of the Saudi Ministry of Health. MERS-CoV infections are identified using real-time, reverse transcription polymerase chain reaction (RT-PCR). The standard assays target amplifications of the upstream E protein (upE gene) and open reading frame (ORF)1a; both need to be positive to confirm infection, otherwise another sample is required to confirm the diagnosis [21]. The sample requires 2 days of processing for the final results to be available.

Routine laboratory testing in our ICU includes complete blood counts, coagulation profile, electrolytes, renal function, liver profile and arterial blood gases. These parameters are measured on admission to the ICU and at least once daily thereafter (at 6:00 am) throughout the ICU stay.

### Management and therapy

All patients with suspected or confirmed MERS-CoV infection were isolated in single rooms, either on the hospital floor or in the ICU. Patients were admitted to the ICU according to the guidelines of the Society of Critical Care Medicine for ICU admission, discharge, and triage [22]. Patients were classified into four categories according to their ICU admission priority: priority one comprised critically ill patients who were unstable and need intensive treatment and monitoring, with significant likelihood of recovery; priority two were stable patients who required intensive monitoring because of the possibility of decompensation; priority three were unstable patients who had a low likelihood of recovery because of the severity of acute disease or because of comorbidities; priority four were those who had little or no anticipated benefit from ICU admission. Patients classified as priority one and two and most of those classified as priority three were admitted to our ICU or full critical care services were mobilized and provided for in the isolation ward until a bed was available in the ICU. Priority four patients were not admitted to the ICU and remained in the isolation ward. General ward patients with MERS-CoV infection were transferred to the ICU if their condition deteriorated or organ failure developed. The infection control precautions recommended by the Saudi Ministry of Health guidelines were strictly implemented to prevent possible transmission of MERS-CoV to other patients or to the healthcare staff [23]. Supportive treatment was provided according to our standard operating procedures and in accordance with the surviving sepsis campaign guidelines [24, 25]. Antiviral therapies, such as oseltamivir, and ribavirin/interferon alfa-2a, were prescribed at the discretion of the attending physician. Protective lung ventilation was applied in mechanically ventilated patients. Prone positioning was considered in some patients with severe refractory hypoxemia. Extracorporeal membrane oxygenation (ECMO) and high-frequency oscillation were also available as a last resort, when considered necessary by the attending physician.

### Statistical analysis

Statistical analyses were performed using SPSS Statistics 19 for Windows (IBM Corp., Armonk, NY, USA). The Kolmogorov-Smirnov test was used to verify whether there were significant deviations from the normality assumption of continuous variables. Nonparametric tests of comparison were used for variables evaluated as not normally distributed. Difference testing between groups was performed using Student’s *t* test, Mann-Whitney test, Chi-square test and Fisher’s exact test, as appropriate. Friedman’s test was used to assess the time course of organ function.

To identify the risk factors for death in the ICU, we performed multivariable logistic regression analyses. Due to the relatively small number of deaths in our study, we adjusted only for the severity of illness on admission to the ICU (APACHE II score) and the degree of organ dysfunction as assessed by admission SOFA score. Potential risk factors for ICU mortality were selected among the demographic characteristics, comorbidities, mode of acquisition of MERS-CoV, initial manifestations, procedures and therapies, and superimposing infections. Variables yielding *P* <0.2 in the univariate analysis, APACHE II score and SOFA score were included in a multivariable logistic regression analysis. These variables were introduced separately into multivariable models including APACHE II and SOFA scores on admission to the ICU. Adjusted odds ratios (OR) and 95 % confidence of interval (CI) were computed. None of the covariates simultaneously introduced in a multivariable model were collinear.

Data are presented as mean ± standard deviation (SD), median value (25th–75th interquartile range [IQR]) or number (%), as appropriate. All statistics were two-tailed and a *P* < 0.05 was considered statistically significant.

## Results

### Characteristics of the study cohort

**Fig. 1:**
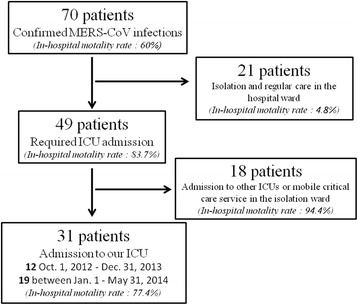
Flow chart representing patients’ inclusion and in-hospital mortality rates according to the admission facility. *ICU* intensive care unit, *MERS-CoV* Middle East respiratory syndrome coronavirus

During the observation period, 70 cases with confirmed MERS-CoV infections were diagnosed in our institution [11] (Fig. 1); 21 patients were managed in the hospital ward, 18 patients were admitted to other ICUs or received critical care service in the ward, and 31 patients were admitted to our ICU (12 between October 1, 2012 and December 31, 2013 and 19 between January 1 and May 31, 2014). Patients were admitted to our ICU because of respiratory failure (PaO2/FiO2 < 250 mmHg).

The mean age of the patients admitted to our ICU (*n* = 31) was 59 (SD 20) years and 22 (71 %) were males. The characteristics of these patients on admission to the ICU are shown in Table 1. Eighteen (58.1 %) patients had community-acquired MERS-CoV infection, while for 13 (47.9 %), including two healthcare staff, infection was acquired in the hospital. Twenty-seven patients (87.1 %) had at least one comorbidity. The median number of concomitant comorbid conditions was three (IQR: 2–4).

**Table 1:** Characteristics of patients on admission to the intensive care unit

	All patients	Nonsurvivors	Survivors	*P* value
N	31	23	8	
Age in years (mean ± SD)	59 ± 20	63 ± 19	46 ± 20	0.048
Male, n (%)	22 (71.0)	15 (65.2)	7 (87.3)	0.379
Body mass index in kg/m^2^ (mean ± SD)	29.8 ± 7.6	29.3 ± 7.1	31.2 ± 9.2	0.707
Severity scores (mean ± SD)				
APACHE II score	24.2 ± 9.5	26.4 ± 8.5	17.8 ± 9.7	0.034
SOFA score	10.7 ± 3.5	11.7 ± 3.1	8.0 ± 3.2	0.01
Acquisition of pneumonia, n (%)				0.689
Community-acquired	18 (58.1)	14 (60.9)	4 (50.0)	
Hospital-acquired	13 (41.9)	9 (39.1)	4 (50.0)	
Comorbidities, n (%)				
Diabetes mellitus	17 (54.8)	13 (56.5)	4 (50.0)	1.00
Arterial hypertension	16 (51.6)	14 (60.9)	2 (25.0)	0.113
Immunosuppressive drugs	7 (22.6)	5 (21.7)	2 (25.0)	1.00
Chronic heart failure	6 (19.4)	4 (17.4)	2 (25.0)	1.00
Chronic kidney disease	6 (19.4)	4 (17.4)	2 (25.0)	0.634
Cerebrovascular accidents	5 (16.1)	4 (17.4)	1 (12.5)	1.00
Coronary artery disease	4 (12.9)	3 (13.0)	0 (0.0)	1.00
Steroid use	3 (9.6)	3 (13.0)	0 (0.0)	0.161
COPD	3 (9.7)	2 (8.7)	1 (12.5)	1.00
Smoking	3 (9.7)	1 (4.3)	2 (25.0)	0.156
Chronic liver disease	3 (9.7)	3 (13.0)	0 (0.0)	0.550
Hypothyroid	3 (9.7)	3 (13.0)	0 (0.0)	0.550
Cancer	2 (6.5)	2 (8.7)	0 (0.0)	1.00
Epilepsy	2 (6.5)	1 (4.3)	1 (12.5)	0.456
Dyslipidemia	1 (3.2)	1 (4.3)	0 (0.0)	1.00
Asthma	1 (3.2)	1 (4.3)	0 (0.0)	1.00
Bronchiectasis	1 (3.2)	1 (4.3)	0 (0.0)	1.00
Obstructive sleep apnea	1 (3.2)	1 (4.3)	0 (0.0)	1.00
Number of comorbidities, median (IQR)	3 (2–4)	3 (2–4)	3 (1–4)	0.580
ICU mortality, n (%)	23 (74.2)	23 (100)	-	NA
ICU length of stay in days [median, (IQR)]	9 (4–16)	10 (4–16)	9 (4–17)	0.912
Hospital length of stay in days [median, (IQR)]	12 (4–16)	10 (4–16)	18 (14–72)	0.002

*APACHE* acute physiology and chronic health evaluation, *COPD* chronic obstructive pulmonary disease, *ICU* intensive care unit, *IQR* interquartile range, *NA* not applicable, *SD* standard deviation, *SOFA* sequential organ failure assessment

### Clinical manifestations

Initial clinical manifestations had occurred at a median of 2 days (IQR: 2–4) prior to hospital admission. Patients had been treated for a median of 5 days (IQR: 2–9) in general hospital wards before their admission to the ICU. Only four patients (12.9 %) were admitted to the ICU on arrival at the hospital. Cough and tachypnea were reported in all patients. Other common initial symptoms were fever (87.1 %), abdominal pain (29 %), sore throat (25.8 %), and fatigue (25.8 %) (Additional file 1). Crackles (93.5 %), tachycardia (67.7 %), and rhonchi (32.3 %) were the most commonly identified initial physical signs. Bilateral pulmonary infiltrates were present in the chest X-rays of 24 (77.4 %) patients and lobar infiltrates in six (19.4 %). Only one patient had a normal chest X-ray at the time of admission to the ICU.

### Microbiology and co-existing infections

On admission to the ICU, no patients had microbiologically proven co-existing bacterial pneumonia. Secondary infections, as evident from positive quantitative cultures of deep tracheal aspirates, occurred in 18 (58.1) patients within a median of 3 days (IQR: 3–8) after admission to the ICU. The most commonly isolated microorganisms were *Acinetobacter baumannii* (25.8 %), *Pseudomonas aeruginosa* (12.9 %) and Candida species (9.7 %) (Additional file 2). Sputum cultures were positive in only four patients. Three patients had evidence of nasal colonization with methicillin-resistant *Staphylococcus aureus* (MRSA) without further microbiologic evidence of bacterial pneumonia.

Only four (12.9 %) patients had positive blood cultures; *Acinetobacter baumannii* (*n* = 2), *Escherichia coli* (*n* = 1), methicillin-resistant *Staphylococcus aureus* (*n* = 1), and vancomycin-resistant *Enterococcus* species (*n* = 1).

### Procedures and therapy

Invasive mechanical ventilation was applied in 27 (87.1 %) patients during the ICU stay; 18 (58.1 %) within 24 hours of admission to the ICU, and 14 (45.5 %) patients received noninvasive ventilation (Table 2). Eleven (35.5 %) patients were treated with high-frequency oscillation and five (16.1 %) with prone positioning. Only one patient received ECMO. The ventilatory parameters are presented in Additional file 3. Vasopressor therapy using norepinephrine was initiated in 25 (80.6 %) patients during the ICU stay, nine (29 %) within 24 hours of ICU admission, and 16 (51.6 %) patients received continuous renal replacement therapy (Table 2). Oseltamivir was administered to 20 (64.5 %) patients for a median of 5 days (IQR: 3–5). Combined ribavirin plus interferon alfa-2a therapy was used in 13 (41.9 %) patients (Table 2). All patients received at least one antimicrobial agent during the ICU stay (Additional file 2). Antifungal therapy was only used in four of the five patients with positive cultures for Candida but the necessity of this therapy is uncertain.

**Table 2:** Procedures and therapies

	All patients	Nonsurvivors	Survivors	*P* value^a^
N	31	23	8	
Noninvasive ventilation, n (%); days, median (IQR)	14 (45.2); 2 (1–3)	8 (34.8); 2 (1–3)	6 (75.0); 2 (1–4)	0.097
Invasive ventilation, n (%); days, median (IQR)	27 (87.1); 8 (4–17)	22 (95.7); 8 (2–17)	5 (62.5); 11 (5–17)	0.043
High-frequency oscillation	11 (35.5)	11 (47.8)	0 (0.0)	0.028
Prone positioning, n (%); days, median (IQR)	5 (16.1); 4 (2–5)	5 (21.7); 4 (2–5)	0 (0.0); 0 (0.0)	1.00
ECMO, n (%); days, median (IQR)	1 (3.2); 13 (13–13)	1 (4.3); 13 (13–13)	0 (0.0); 0 (0.0)	1.00
Vasopressors^b^, n (%); days, median (IQR)	25 (80.6); 4 (2–9)	22 (95.7); 5 (2–9)	3 (37.5); 3 (2–3)	0.002
Intermittent hemodialysis, n (%); days, median (IQR)	3 (9.7); 5 (2–5)	2 (8.7); 4 (2–4)	1 (12.5); 7 (7–7)	1.00
Continuous RRT, n (%); days, median (IQR)	16 (51.6); 6 (2–11)	14 (60.9); 7 (3–11)	2 (25.0); 2 (1–2)	0.113
Medications, n (%)				
Oseltamivir	20 (64.5)	15 (65.2)	5 (62.5)	0.484
Ribavirin/interferon alfa-2a	13 (41.9)	9 (39.1)	4 (50.0)	0.507
Neuromuscular blockers	12 (38.7)	10 (43.5)	2 (25.0)	0.465
Steroids^c^	8 (25.8)	6 (26.1)	2 (25.0)	0.954

*ECMO* extracorporeal membrane oxygenator, *IQR* interquartile range, *RRT* renal replacement therapy

^a^Comparisons done for frequencies

^b^Norepinephrine

^c^Intravenous hydrocortisone (200–300 mg per day) in four patients and methyprednisolone in four patients (maintenance therapy for underlying disease)

### Morbidity and mortality

The overall ICU mortality rate was 74.2 % (*n* = 23). The median ICU and hospital lengths of stay were 9 (IQR: 4–16) and 12 (IQR: 4–16) days, respectively. The major causes of death were hypoxemic respiratory failure (52.2 %) and refractory septic shock (26.1 %). One patient died from sudden cardiac arrest after ICU discharge but while still in the hospital. Furthermore, one patient died within 1 year after discharge from the ICU because of septic shock related to an infected wound. Only one patient was lost to follow-up after hospital discharge.

**Fig. 2:**
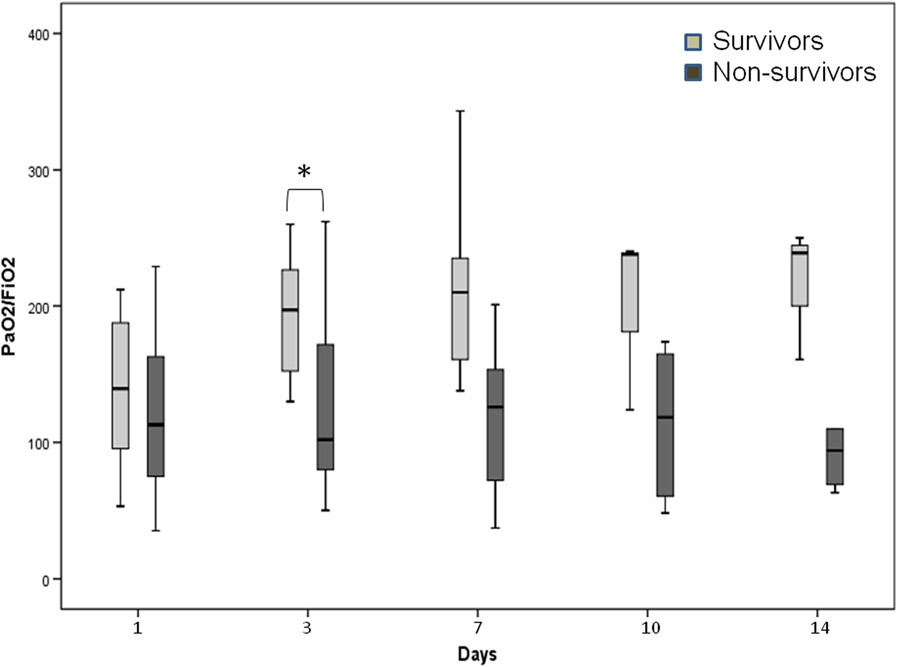
Box plot representing the time course of PaO2/FiO2 according to ICU outcome. *P < 0.005 compared to survivors

The SOFA score and Glasgow Coma Scale (GCS) increased markedly over the first 2 weeks in the ICU in the whole cohort, while other parameters of organ function remained largely unchanged (Additional file 3). Compared with those who were discharged alive from the ICU, nonsurvivors were older, had higher APACHE II and SOFA scores on admission to the ICU, and were more likely to require invasive mechanical ventilation and vasopressor therapy and to have been ventilated using high-frequency oscillation (Tables 1 and 2, and Additional files 1 and 2). Nonsurvivors had a persistently low PaO2/FiO_2_ throughout the first 2 weeks in the ICU, whereas survivors showed a slight increase over time (Fig. 2). After adjustment for the severity of illness and the degree of organ dysfunction, the need for vasopressors was the only independent risk factor for death in the ICU (OR 18.33, 95 % confidence interval 1.11–302.1, *P* 0.04) (Additional file 4).

## Discussion

The 31 critically ill patients with confirmed MERS-CoV infection in our cohort frequently had organ failure with an overall mortality rate greater than 74 %. Comorbidities were common in this cohort of patients. Not surprisingly, mortality in the ICU was associated with older age, severe disease and organ failure. The need for vasopressor therapy was an independent risk factor of death in the ICU.

Since the first reported case of MERS-CoV infection in 2012, several authors have described various cohorts of patients with this serious infection [8, 10–12, 14, 15, 26]. Most of the reports included all microbiologically confirmed cases, irrespective of the severity of illness and the clinical course. Indeed, the clinical features of MERS-CoV infection range from asymptomatic or mild disease to acute respiratory distress syndrome (ARDS) and multi-organ failure [13, 27]. The most severe cases, which require admission to the ICU, are potentially life-threatening and represent a major challenge to the healthcare system. To date, only three studies have reported the clinical characteristics and outcomes of patients with MERS-CoV infection who were admitted to the ICU; collectively including a total of only 34 patients [16–18]. Arabi et al. included 12 patients admitted to two ICUs in Riyadh and one in Al-Hasa in the central and eastern parts of the country, respectively [17]. The other two reports were both from Jeddah, western Saudi Arabia. Al-Hameed et al. included eight patients, whereas Khalid et al. described the clinical course and outcomes of 14 patients with severe MERS-CoV infection and ARDS [16, 18]. Our facility is a large tertiary-care medical center in Riyadh, central Saudi Arabia. We herein provide a detailed account of the largest single cohort of critically ill MERS-CoV infected patients reported thus far.

In agreement with previous reports from Saudi Arabia, comorbid conditions were common in our patients with MERS-CoV infections with a median of three comorbidities per patient [10, 11, 15, 17]. In contrast, only 54.8 % of the 186 individuals involved in the recent MERS-CoV outbreak in South Korea had any preexisting chronic medical conditions [8]. However, only 29.6 % of patients in the Korean outbreak were aged 65 years or older and nearly half (46.2 %) were caregivers or healthcare personnel [8]. The differences in the demographic characteristics of our cohorts and the mode of acquisition of MERS-CoV infection may explain, at least in part, the discrepancy in the patterns of associated comorbidities between the Saudi and Korean cohorts.

The respiratory manifestations of MERS-CoV infection in our cohort were similar to those observed in previous reports from Saudi patients [10, 11, 14, 15, 17]. Cough and tachypnea occurred in all patients and 77 % of cases had bilateral pulmonary infiltrates, denoting severe respiratory illness, which required a median of 5 days to reach the peak of clinical deterioration such that ICU admission and organ support therapy were required. Gastrointestinal manifestations, such as abdominal pain, diarrhea, vomiting, and abdominal tenderness, were relatively common in our cohort. This was also a common finding in the previous literature in patients with MERS-CoV infection as well as those with severe acute respiratory syndrome (SARS) [10, 11, 15, 17, 28, 29].

Our data confirm previous studies that reported a high prevalence of nonrespiratory organ failure in critically ill patients with MERS-CoV [16, 17]. The mechanisms of organ dysfunction and failure in these patients are yet to be determined. Cytokine dysregulation has been suggested to be involved in the pathophysiology of MERS-CoV-related organ failure. Direct viral invasion may also occur as the virus was recovered from urine and stool in one patient [30]. In agreement with the results of the previous reports on critically ill patients with MERS-CoV infection [16–18], more than 80 % of our patients received vasopressor support, underscoring the high prevalence of cardiovascular dysfunction in these patients, and suggesting that disturbances in tissue perfusion may also have been involved in the pathophysiology of the organ failure. Lower rates of vasopressor support have been reported in patients with SARS [10, 11, 15, 17, 28, 29] with, as a result, lower mortality rates than those reported in patients with MERS-CoV infections.

Even though overall mortality rate was high in our cohort, it is still comparable with rates reported in previous studies (58.3–64.3 %) [16–18]. In all studies, almost all patients had significant comorbidities and median APACHE II scores of 25 or higher. We observed significantly higher APACHE II and SOFA scores in ICU nonsurvivors compared to those who survived severe MERS-CoV infection, underscoring the strong association between mortality and the severity of disease.

Epidemiological analyses have suggested that MERS-CoV is unlikely to trigger sustained human epidemics at present [31, 32]. Nevertheless, nosocomial outbreaks have resulted in considerable morbidity and mortality, in addition to disruption of medical services and substantial economic losses [9, 33, 34]. The most severe infections usually require ICU admission, necessitate major resource utilization and result in high fatality rates. Identifying possible risk factors for poor prognosis in patients with MERS-CoV infection is therefore crucial to enable appropriate allocation of healthcare resources and early transfer of high-risk patients to the appropriate medical facilities. Our data show that the need for vasopressor therapy was an independent risk factor for death in the ICU. Indeed, the major causes of death in our study were hypoxemic respiratory failure and refractory septic shock, which confirm the role of respiratory and cardiovascular system failures as determinants of outcome in this population. This was also evident from the persistent hypoxemia observed in the nonsurvivors. To date, published data on the risk factors for poor prognosis specific to critically ill patients with MERS-CoV infection are lacking. In cohort studies of patients with any degree of severity of MERS-CoV infection, older age, diabetes, chronic renal failure, chronic respiratory disease, high viral load in lower respiratory tract samples, shorter incubation period and MERS-CoV viremia have all identified as independent predictors of mortality [11, 14, 35–37].

Secondary respiratory infections occurred commonly in this cohort, predominantly with Gram-negative bacteria. Although Candida species were frequently isolated, these are probably not relevant as respiratory pathogens and the necessity of antifungal therapy is uncertain. Interestingly, *Acinetobacter baumannii*, which is an emerging fatal infection in ICU patients worldwide, was isolated from deep tracheal aspirates in one in four patients. This may explain, at least in part, the relatively high mortality rates in this cohort.

Specific therapeutic options for MER-CoV infections are limited and their efficacy is not well established [38]. All patients in this report received antiviral treatment with either oseltamivir or combined ribavirin/interferon alfa-2a therapy; two patients received both. Although a previous study from the same institution showed that combined ribavirin/interferon alfa-2a therapy was associated with significant improvement in survival at 14 days, this benefit was not maintained at 28 days after the onset of the disease [39]. The retrospective and observational nature of this study does not allow precise assessment of the efficacy of these therapies. In the absence of a vaccine or a specific treatment, prevention of viral transmission through adequate infection control methods is the mainstay in the management of MERS-CoV outbreaks. Appropriate isolation of patients with suspected or proven infections is crucial. In view of the high fatality rates of these patients in the ICU, it may be reasonable to closely monitor patients with suspected infections in the general wards for early signs of organ dysfunction to prevent unnecessary delay in the provision of intensive care services and reduce mortality rates in these patients.

Our study has some limitations. We included patients with confirmed MERS-CoV infection from one ICU of a large medical center. Possible variations in the geographic distribution of the disease and in local practice may hinder extrapolation of these data to other cohorts in Saudi Arabia and other countries. The relatively low number of patients may have biased the statistical comparisons presented in this report and overestimated mortality rates. Multivariable adjustment was also limited to the variables included in the models. Collaborative efforts are needed to provide an insight into the risk factors for poor prognosis in these patients.

## Conclusions

MERS-CoV infections requiring admission to the ICU are associated with high morbidity and mortality rates. The need for vasopressor therapy is the main risk factor for death in these patients.

## Key messages

- This report describes the clinical features and outcomes of 31critically ill patients with confirmed Middle East respiratory syndrome coronavirus (MERS-CoV) infection.
- Patients with MERS-CoV infections frequently had organ failure, and mortality rates were greater than 72 %.
- The need for vasopressor therapy was an independent risk factor for death in the ICU.

## Additional files

Additional file 1: A table presenting the initial clinical manifestations in patients with MERS-CoV infections. (DOCX 27 kb) Additional file 2: A table presenting the superimposed respiratory tract infections and antibiotic use. (DOCX 28 kb) Additional file 3: A table presenting the time course of organ function parameters in the whole cohort. (DOCX 28 kb) Additional file 4: A table presenting the crude and adjusted odds ratios of death in the ICU among the possible risk factors. (DOCX 26 kb)

## Abbreviations

APACHE: acute physiology and chronic health evaluation score
ARDS: acute respiratory distress syndrome
CI: confidence interval
ECMO: extracorporeal membrane oxygenation
GCS: Glasgow Coma Scale
ICU: intensive care unit
IQR: interquartile range
MERS-CoV: Middle East respiratory syndrome coronavirus
OR: odds ratio
ORF: open reading frame
RT-PCR: reverse transcription polymerase chain reaction
SARS: severe acute respiratory syndrome
SD: standard deviation
SOFA: sequential organ failure assessment
upE: upstream E protein
