# Supplementary material for: Presentation and outcome of Middle East respiratory syndrome in Saudi intensive care unit patients
Source: Crit Care. 2016 May 7;20:123. doi: 10.1186/s13054-016-1303-8 (PMC4859954; doi:10.1186/s13054-016-1303-8)
Supplement: Additional file 1: — A table presenting the initial clinical manifestations in patients with MERS-CoV infections. (DOCX 27 kb) [file 13054_2016_1303_MOESM1_ESM.docx]

Additional file 1. Initial clinical manifestations

|  | All patients | Non-survivors | Survivors | *P* value |
| --- | --- | --- | --- | --- |
| N | 31 | 23 | 8 |  |
| Onset of symptoms prior to ICU admission, days, median (IQR)* | 2 ((-2)-4) | 0 ((-3)-3) | 3 ((-6)-6) | 0.317 |
| Pre-admission LOS, days, median (IQR) | 5 (2-9) | 5 (3-9) | 3 (1-8) | 0.255 |
| Initial symptoms, n (%) |  |  |  |  |
| Cough | 31 (100) | 23 (100) | 8 (100) | 0.583 |
| Fever | 27 (87.1) | 20 (87.0) | 7 (87.5) | 1.00 |
| Abdominal pain | 9 (29.0) | 7 (30.4) | 2 (25.0) | 1.00 |
| Sore throat | 8 (25.8) | 6 (26.1) | 2 (25.0) | 1.00 |
| Fatigue | 8 (25.8) | 5 (21.7) | 3 (37.3) | 0.393 |
| Mental changes | 7 (22.6) | 6 (26.1) | 1 (12.5) | 0.642 |
| Chills | 6 (19.4) | 4 (17.4) | 2 (25.0) | 0.643 |
| Myalgia | 6 (19.4) | 3 (13.0) | 3 (37.3) | 0.161 |
| Diarrhea | 6 (19.4) | 4 (17.4) | 2 (25.0) | 0.634 |
| Hemoptysis | 5 (16.1) | 3 (13.0) | 2 (25.0) | 1.00 |
| Chest pain | 5 (16.1) | 4 (17.4) | 1 (12.5) | 1.00 |
| Vomiting | 4 (12.9) | 4 (17.4) | 0 (0.0) | 0.550 |
| Runny nose | 3 (9.7) | 1 (4.3) | 2 (25.0) | 0.156 |
| Signs, n (%) |  |  |  |  |
| Tachypnea | 31 (100) | 23 (100) | 8 (100) | 1.00 |
| Crackles | 29 (93.5) | 23 (100) | 6 (75.0) | 0.06 |
| Tachycardia | 21 (67.7) | 17 (73.9) | 4 (50.0) | 0.381 |
| Fever | 19 (61.3) | 15 (65.2) | 4 (50.0) | 0.616 |
| Rhonchi | 10 (32.3) | 6 (26.1) | 4 (50.0) | 0.381 |
| Abdominal tenderness | 4 (12.9) | 4 (17.4) | 0 (0.0) | 0.550 |
| Convulsions | 3 (9.7) | 1 (4.3) | 2 (25.0) | 0.156 |
| Bronchial breathing | 2 (6.5) | 2 (8.7) | 0(0.0) | 1.00 |
| Skin rash | 2 (6.5) | 2 (8.7) | 0 (0.0) | 1.00 |
| Physiologic derangements, n (%)† |  |  |  |  |
| Leukopenia | 4 (12.9) | 1 (4.3) | 3 (37.5) | 0.043 |
| Lymphopenia | 26 (83.9) | 19 (82.6) | 7 (87.5) | 1.00 |
| Thrombocytopenia | 11 (35.5) | 8 (34.8) | 3 (37.5) | 1.00 |
| Initial chest x-ray finding, n (%) |  |  |  | 0.564 |
| Bilateral infiltrates | 24 (77.4) | 18 (78.3) | 61 (75.0) |  |
| Lobar infiltrates | 6 (19.4) | 4 (17.4) | 2 (25.0) |  |
| Normal | 1 (3.2) | 1 (4.3) | 0 (0.0) |  |

IQR, interquartile range; LOS, length of stay

*In relation to hospital admission.

†Leukopenia: white blood cell count < 4 x10^9^ cells/L, lymphopenia: lymphocytes < 1.5 x10^9^ cells/L, and thrombocytopenia <130 x10^9^ cells/L.
