# Supplementary material for: Presentation and outcome of Middle East respiratory syndrome in Saudi intensive care unit patients
Source: Crit Care. 2016 May 7;20:123. doi: 10.1186/s13054-016-1303-8 (PMC4859954; doi:10.1186/s13054-016-1303-8)
Supplement: Additional file 2: — A table presenting the superimposed respiratory tract infections and antibiotic use. (DOCX 28 kb) [file 13054_2016_1303_MOESM2_ESM.docx]

Additional file 2. Superimposed respiratory tract infections and antibiotic use

|  | All patients | Non-survivors | Survivors | *P* value |
| --- | --- | --- | --- | --- |
| N | 31 | 23 | 8 |  |
| Positive respiratory cultures, n (%) | 18 (58.1) | 13 (56.5) | 5 (62.5) | 0.464 |
| - Tracheal aspirates or BAL* | 11 (35.5) | 8 (34.8) | 3 (37.5) | 1.000 |
| *Acinetobacter baumannii* | 8 (25.8) | 5 (16.1) | 3 (37.5) | 0.393 |
| *Pseudomonas aeruginosa* | 4 (12.9) | 2 (8.7) | 2 (25.0) | 0.268 |
| Candida species | 3 (9.7) | 1 (4.3) | 2 (25.0) | 1.000 |
| *Klebsiella pneumoniae* | 1 (3.2) | 1 (4.3) | - | 1.000 |
| - Sputum | 4 (12.9) | 2 (8.7) | 2 (25.0) | 0.268 |
| *Acinetobacter baumannii* | 1 (3.2) | 1 (4.3) | - | 1.000 |
| *Pseudomonas aeruginosa* | 1 (3.2) | 1 (4.3) | - | 1.000 |
| Candida species | 2 (6.5) | 1 (4.3) | 1 (12.5) | 0.456 |
| - Nasal swabs | 3 (9.7) | 2 (8.7) | 1 (12.5) | 1.000 |
| MRSA | 3 (9.7) | 2 (8.7) | 1 (12.5) | 1.000 |
| Positive blood cultures, n (%)† | 4 (12.9) | 4 (17.4) | 0 (0.0) | 0.550 |
| Antibiotics, n (%) |  |  |  |  |
| Penicillins | 14 (45.2) | 11 (47.8) | 3 (37.7) | 0.698 |
| Cephalosporins | 1 (3.2) | 1 (4.3) | - | 1.000 |
| Other ß lactam | 20 (64.5) | 15 (65.2) | 5 (62.5) | 1.000 |
| Macrolides | 11 (35.5) | 8 (34.8) | 3 (37.5) | 1.000 |
| Glycopeptides | 17 (54.8) | 13 (56.5) | 4 (50) | 1.000 |
| Quinolones | 3 (9.7) | 2 (8.7) | 1 (12.5) | 1.000 |
| Other antibacterials | 18 (58.1) | 14 (60.9) | 4 (50) | 0.689 |
| Antifungal | 4 (12.9) | 3 (13.0) | 1 (12.5) | 1.000 |

* Only 1 patient with positive BAL for candida species with concomitantly positive culture of tracheal aspirate with *Acinetobacter baumannii* and *Pseudomonas aeruginosa*.

† Confirmed on at least two sets of blood cultures.

BAL, broncholaveolar lavage; MRSA, methicillin-resistent *Staphylococcus aureus*.
