# Supplementary material for: Presentation and outcome of Middle East respiratory syndrome in Saudi intensive care unit patients
Source: Crit Care. 2016 May 7;20:123. doi: 10.1186/s13054-016-1303-8 (PMC4859954; doi:10.1186/s13054-016-1303-8)
Supplement: Additional file 3: — A table presenting the time course of organ function parameters in the whole cohort. (DOCX 28 kb) [file 13054_2016_1303_MOESM3_ESM.docx]

Additional file 3. Time course of organ function parameters in the whole cohort (n=31)*

|  | Day 1 | Day 3 | Day 7 | Day 10 | Day 14 | *P* value† |
| --- | --- | --- | --- | --- | --- | --- |
| N | 31 | 29 | 21 | 15 | 12 |  |
| Glasgow Coma Scale, points | 11 (8-15) | 7 (3-13) | 6 (3-9) | 6 (3-9) | 3 (3-9) | 0.002 |
| Heart rate, beats/min | 103 (87-123) | 95 (89-110) | 102 (97-116) | 100 (76-118) | 108 (72-116) | 0.809 |
| Mean arterial pressure, mmHg | 62 (52-72) | 60 (55-70) | 65 (57-71) | 61 (57-71) | 61 (53-69) | 0.505 |
| PaO_2_/FiO_2_, mmHg | 118 (73-166) | 130 (91-210) | 140 (81-161) | 124 (71-172) | 103 (72-176) | 0.929 |
| Creatinine, µmol/L‡ | 127 (74-267) | 157 (100-282) | 141 (98-177) | 162 (129-242) | 151 (85-313) | 0.439 |
| CK, IU/L§ | 112 (50-360) | 176 (90-580) | 284 (125-734) | 130 (54-1391) | 177 (69-1321) | 0.194 |
| ALT, IU/L‖ | 35 (20-55) | 38 (22-77) | 48 (26-94) | 47 (37-59) | 45 (30-70) | 0.821 |
| AST, U/L¶ | 61 (40-100) | 65 (43-158) | 55 (38-131) | 65 (47-140) | 88 (54-185) | 0.742 |
| Bilirubin, µmol/L¶ | 11 (5-24) | 17 (9-39) | 16 (11-28) | 18 (10-45) | 31 (14-43) | 0.094 |
| WBC x10^9^ cells/L** | 9.7 (6.3-12.6) | 8.6 (5.8-12.2) | 12.5 (9.5) | 11.0 (8.5-12.8) | 18.0 (15.3-20.0) | 0.05 |
| Platelets x10^9^ cells/L†† | 165 (129-265) | 152 (120-233) | 177 (106-272) | 164 (55-270) | 185 (121-296) | 0.670 |
| SOFA score, points | 11 (8-14) | 12 (10-15) | 13 (11-15) | 14 (10-16) | 13 (11-17) | 0.022 |
| Ventilatory settings |  |  |  |  |  |  |
| Tidal volume, mL/Kg | 5.4 (4.2-6.0) | 5.2 (4.6-6.1) | 4.9 (4.0-5.3) | 4.8 (3.8-6.0) | 5.5 (3.9-6.1) | 0.736 |
| Plateau pressure, cmH_2_O | 28 (25-31) | 28 (27-32) | 28 (24-31) | 30 (24-33) | 27 (22-31) | 0.774 |
| PEEP, cmH_2_O | 12 (8-14) | 10 (10-14) | 10 (8-12) | 10 (8-12) | 10 (9-12) | 0.260 |

ALT, alanine aminotransferase; AST, aspartate aminotransferase; CK, creatine kinase; PEEP, positive end expiratory pressure; SOFA, sequential organ failure assessment; WBC, white blood cell count.

*Data presented as median (interquartile range).

†Comparisons between groups using Friedman’s Test.

‡Normal range, 50-98 µmol/L.

§Normal range, 50-170 IU/L

‖Normal range, 5-55 U/L

¶Normal range 5-34 U/L

**Normal range 4-11 x10^9^ cells/L

††Normal range 150-400 x10^9^ cells/L
