# Supplementary material for: Presentation and outcome of Middle East respiratory syndrome in Saudi intensive care unit patients
Source: Crit Care. 2016 May 7;20:123. doi: 10.1186/s13054-016-1303-8 (PMC4859954; doi:10.1186/s13054-016-1303-8)
Supplement: Additional file 4: — A table presenting the crude and adjusted odds ratios of death in the ICU among the possible risk factors. (DOCX 26 kb) [file 13054_2016_1303_MOESM4_ESM.docx]

Additional file 4. Crude and adjusted* odds ratios of death in the ICU among the possible risk factors (*P* < 0.2 in a univariate logistic regression analysis).

|  | Odds ratio (95% confidence interval) | | | |
| --- | --- | --- | --- | --- |
|  | Crude | p-value | Adjusted* | *P* value |
| N |  |  |  |  |
| Age (per year) | 1.05 (1-1.09) | 0.05 | 1.01 (0.95-1.08) | 0.677 |
| Comorbidities |  |  |  |  |
| Arterial hypertension | 4.77 (0.77-28.4) | 0.095 | 1.77 (0.15-20.43) | 0.594 |
| Steroids | 0.25 (0.04-1.63) | 0.148 | 0.12 (0.01-1.81) | 0.127 |
| Smoking | 7.33 (0.56-95.29) | 0.128 | 7.55 (0.26-226.27) | 0.238 |
| Initial manifestations |  |  |  |  |
| Myalgia | 0.25 (0.04-1.63) | 0.148 | 0.19 (0.018-1.93) | 0.159 |
| Runny nose | 0.14 (0.01-1.77) | 0.128 | 0.2 (0.01-5.87) | 0.352 |
| Leukopenia | 0.08 (0.01-0.89) | 0.04 | 0.024 (0.01-1.31) | 0.067 |
| Procedures |  |  |  |  |
| Non-invasive ventilation | 0.41 (0.05-3.34) | 0.062 | 0.18 (0.03-1.09) | 0.406 |
| Invasive ventilation | 13.2 (0.13-154.9) | 0.04 | 4.58 (0.28-76.11) | 0.288 |
| Continuous RRT | 4.67 (0.77-28.41) | 0.095 | 2.4 (0.31-18.49) | 0.397 |
| Need for vasopressors | 36.67 (3.12-430.33) | 0.004 | 18.33 (1.11-302.1) | 0.042 |

RRT, renal replacement therapy.

*Adjusted for APACHE II and SOFA scores on admission to the ICU in a multivariable logistic regression analysis with ICU mortality as the dependent varaiable. Each listed covariate was introduced separately in a multivariable model with the severity scores.
